# Population-Based Genetic Assessment of Thrombophilia Polymorphisms: Allelic Frequencies and Population Linkage Dynamics

**DOI:** 10.3390/medicina61111935

**Published:** 2025-10-29

**Authors:** Panagiota Tsiatsiou, Sofia Balaska, Zafeirios Tsinaris, Maria Archonti, Antonia Lanta, Vasiliki Tsaireli, Anna Takardaki, Areti Kourti, Angeliki Kassomenaki, Georgios Meletis, Dimitrios A. Tsakiris, Lemonia Skoura

**Affiliations:** 1 Laboratory Hematology, Microbiology Department, AHEPA University Hospital, 54636 Thessaloniki, Greece; 2 Laboratory of Biochemistry, AHEPA University Hospital, School of Medicine, Aristotle University of Thessaloniki, 54636 Thessaloniki, Greece; 3 School of Medicine, Faculty of Health Sciences, Aristotle University of Thessaloniki, University Campus, 54124 Thessaloniki, Greece

**Keywords:** thrombophilia, allelic frequencies, Hardy–Weinberg equilibrium, linkage disequilibrium

## Abstract

*Background and Objectives*: Thrombophilia is a prothrombotic disorder that increases the risk of blood clotting and can pose serious health problems. It is considered a condition of gene–gene or gene–environment interactions. Variation in the prevalence of thrombophilia mutations and their interaction among populations necessitates localized genetic assessments. However, population-based genetic data remains limited for developing effective preventive strategies. *Materials and Methods*: This cross-sectional observational study was conducted over five years (2020–2024) at a tertiary university hospital in Northern Greece. A total of 2961 individuals aged 18–85 years (mean: 50.5) were registered based on family or medical history of venous thromboembolism (VTE) or clinical symptoms of VTE. The final analysis included 2078 participants comprising 1143 males (55%) and 935 females (45%), who met all the inclusion criteria. Inclusion criteria were absence of acute illness or malignancy, informed consent, and an adequate DNA quantity for genotyping, whereas excluded criteria included incomplete laboratory data, active inflammatory or malignant disease, and cognitive or psychiatric conditions. Peripheral blood samples were collected in 2 mL K_3_-EDTA tubes, and genomic DNA was analyzed using real-time polymerase chain reaction (PCR) with melting curve analysis and hybridization probes (LightMix^®^ in vitro diagnostics, TIB MolBiol, Berlin, Germany). Five thrombophilia-related polymorphisms, Factor V Leiden (*F5* G1691A), prothrombin (*F2* G20210A), methylenetetrahydrofolate reductase (*MTHFR* C677T and *MTHFR* A1298C), and Plasminogen Activator Inhibitor-1 (*PAI-1*) 4G/5G, were examined for allele and genotype frequencies, Hardy–Weinberg equilibrium testing, pairwise linkage disequilibrium (D′ and r^2^), and power analysis. For subjects tested for Factor V Leiden (*n* = 1476), the activated protein C resistance (APC) ratio was additionally evaluated using the ACL TOP 750 analyzer. *Results*: Allele frequencies were 7.3% for FV Leiden and 3.7% for *FII*. The *PAI-1* allele was distributed at 44%, while the *MTHFR* (C677T and A1298C) alleles were each present at 33%. Significant linkage disequilibrium was identified between *MTHFR* (C677T and A1298C) and between *MTHFR* A1298C and *PAI-1*. No evolutionary pressure or demographic bias was found in the Hardy–Weinberg equilibrium. The APC ratio demonstrated a high sensitivity (99.2%) and specificity (96.6%), indicating that it may serve as a reliable screening method. *Conclusions*: Our findings highlight informative patterns in the genetic predisposition to thrombophilia, which may help develop rule-based strategies for implementing thromboprophylaxis guidelines and personalized medical interventions.

## 1. Introduction

Thrombophilia is defined by an increased tendency to form blood clots, which may result in severe complications such as pulmonary embolism and venous thromboembolism. Genetic and acquired factors influence the risk of developing thrombophilia and its associated complications. Key polymorphisms, such as Factor V Leiden (FVL), prothrombin G20210A mutation (*FII*), methylenetetrahydrofolate reductase (*MTHFR* C677T and *MTHFR* A1298C) polymorphisms, and plasminogen activator inhibitor-1 (*PAI-1*), play a crucial role in thrombophilia, when present alone or in combination.

FV Leiden (*FV* G1691A), the most common type of inherited thrombophilia, is caused by a single nucleotide substitution in the *F5* gene. This mutation, rs6025, replaces arginine with glutamine at position 506 in the factor V protein, altering the activated protein C (APC) cleavage site at Arg506. This mutation confers a 3- to 8-fold relative increase in the risk of thrombosis [1,2].

The *FII* mutation is a single-nucleotide change, a genetic variation in which the nucleotide guanine (G) is replaced with adenine (A) at position 20210. The mutation is a weak yet significant risk factor for thromboembolism, and its impact is amplified when combined with other genetic (e.g., FVL) or acquired (e.g., surgery, immobility, and diabetes) factors [3,4,5,6,7].

The *MTHFR* C677T (rs1801133) and A1298C (rs1801131) polymorphisms are variants of the methylenetetrahydrofolate reductase (*MTHFR*) gene, which is crucial for the regulation of homocysteine and folate metabolism. The C677T mutation substitutes alanine with valine, resulting in a 30% decrease in enzymatic activity, whereas the A1298C mutation replaces glutamate with alanine, causing a milder reduction [8,9].

Plasminogen activator inhibitor-1 (*PAI-1*), a serine protease inhibitor of tissue-type and urokinase-type plasminogen activators (t-PA and u-PA), regulates fibrinolysis by preventing plasmin generation and subsequent fibrin degradation. Elevated *PAI-1* levels are associated with an increased risk of venous and arterial thrombotic disease [10,11]. *PAI-1* promoter gene polymorphism 4G/5G has been correlated with high *PAI-1* levels and arterial thrombosis [12,13,14].

The prevalence of mutations related to thrombophilia varies significantly between populations. In previous studies in Greece, factor V Leiden allele frequencies ranged from about 2.5% to 7.5%, Prothrombin G20210A frequencies ranged from 2.2% to 4.5%, and *MTHFR* C677T allele frequencies ranged from 35.3% to 54.6%. These values are among the highest reported in Europe, where the mean frequency of the FV Leiden mutation is 2.7–4.4%, with a north-to-south gradient that peaks in Mediterranean countries. Thus, the *F2* G20210A allele is distinctly more prevalent in Southern Europe at 1.3–2.2% compared to Central and Northern Europe at 0.7–2%. The *MTHFR* C677T variant is present in up to 45% of the population worldwide, depending on ethnicity [15,16,17,18].

In our study, the selected variants are among the most clinically relevant and well-characterized genetic determinants of thrombophilia, collectively influencing coagulation, homocysteine metabolism, and fibrinolysis. These genetic variants collectively represent the main determinants of hereditary and multi-factorial thrombophilia, allowing the simultaneous evaluation of procoagulant and hypofibrinolytic pathways in the same population. Being both highly prevalent and extensively investigated, they are among the most common thrombophilia-related polymorphisms in European populations and serve as key markers for laboratory-based risk assessment and comparative population studies in Greece.

Variability in national guidelines for thrombophilia screening limits the scope of large-scale population-based genetic studies, resulting in selective testing and underrepresentation of specific groups rather than comprehensive, systematic approaches. Using real-time PCR, this study aimed to estimate the allele and genotype frequencies of thrombophilia polymorphisms, specifically FVL, *FII*, *MTHFR* (C677T, A1298C), and *PAI-1*, to understand their distribution within a population-based area and compare these findings with those of other European and global populations. Additionally, this study explored linkage disequilibrium patterns to examine the co-inheritance of polymorphisms and assess the Hardy–Weinberg equilibrium to determine whether population structures remain constant across generations. Also, the research investigated the correlation between the FVL genotype distribution (wild-type, heterozygous, and homozygous mutant) and the activated protein C (APC) ratio. Power analysis was conducted to ensure the robustness of the genetic study.

High-quality population-based data help clarify which subgroups benefit most from genetic testing, as well as aid in understanding the regional prevalence patterns of thrombophilia, especially in similar ancestral backgrounds. This information is crucial for understanding and applying rule-based approaches to thromboprophylaxis recommendations, which helps to track health trends and individual treatment plans.

## 2. Materials and Methods

### 2.1. Study Design and Data

A cohort of 2961 individuals aged 18–85 years (mean: 50.5) was enrolled in a cross-sectional observational study. Over five years (2020–2024), the study surveyed several regions in Northern Greece. The participants were patients and outpatients at a tertiary hospital (AHEPA) who were chosen for thrombophilia screening by their physicians based on either a family or medical history of venous thromboembolism (VTE) or the presence of clinical VTE symptoms. After applying the inclusion and exclusion criteria, the final analysis included 2078 participants, comprising 1143 males (55%) and 935 females (45%).

The survey population was recruited from northern Greece, including both urban and rural areas, to minimize potential heterogeneity and stratification biases. This approach ensures a demographically stable and ethnically homogeneous population study, suitable for accurate estimation of allele frequencies and linkage parameters. Although region-specific, this sampling framework provides a reliable genetic baseline for subsequent national and inter-European comparative analyses.

The criteria for inclusion in the study were the absence of acute disease or malignancy at the time of sampling, the provision of informed consent, and sufficient DNA quantity for genotyping. Exclusion criteria were individuals with insufficient laboratory data, acute inflammation or malignant diseases, or cognitive or mental illnesses that have prevented understanding the process. Participants who did not provide consent or whose understanding of consent information was uncertain were excluded from the study to ensure compliance with ethical and legal standards. Age and sex were recorded for all participants, as both variables can influence the apparent distribution and clinical expression of thrombophilia-associated alleles, through hormonal effects and age-related changes in coagulation physiology. Hospital electronic medical records were used to collect data.

Hematologists and internal medicine specialists refer patients for testing under standardized diagnostic criteria, minimizing biases in referrals. To reduce variables that could interfere with results, patients receiving simultaneous anticoagulant therapy at the time of the sampling, pregnant, hormone use, or acute infection are systematically identified and excluded according to medical records and patient history. Regional ancestors were homogeneous (North Greece), reducing the stratification effect on the distribution of the alleles. The study was approved by the AHEPA Hospital Research Ethics Committee (569/12-2024). The Helsinki Declaration adhered to all procedures conducted on the patients.

### 2.2. Genetic Analysis

We conducted a comprehensive genetic analysis of five polymorphic markers: factor V Leiden (*F5*), Prothrombin G20210A (*F2*), methyltetrahydrofolate reductase, *MTHFR* C677T (*MTHFR*_677), methyltetrahydrofolate reductase, *MTHFR* A1298C (*MTHFR*_1298), and methyl-activator inhibitor 1 (*PAI-1*). Peripheral blood samples were collected in 2 mL tubes with K_3_-EDTA. The genomic DNA was extracted using manufacturer standard protocols and high-purity PCR template preparation kits (Roche Molecular Biochemicals, Mannheim, Germany). Spectrophotometry (NanoDrop 2000, Thermo Fisher Scientific, Waltham, MA, USA) was employed to confirm the concentration and purity of DNA. The DNA samples were analyzed in five polymorphisms with real-time PCR, hybridization probes, and melting curve analysis (LightMix^®^, TIB MolBiol, Berlin, Germany). The melting temperature (Tm) is the precise temperature at which half of the DNA molecule becomes single-stranded. Each run includes one heterozygous control (HT), both genotyping standards of wild type (WT) and mutant type (MT), and one No-Target Control (NTC). Also, external quality assessment schemes (RFB, Bonn, Germany) were evaluated to ensure compliance with international molecular diagnostic standards. According to manufacturer validation data and standard molecular diagnostics, internal evaluations were carried out to assess analytical performance features such as sensitivity, specificity, and reproducibility. DNA molecules with different sequences exhibited distinct Tm values.

In subjects in whom FVL testing was performed, we additionally evaluated the activated protein C ratio (APC) in homologous plasma samples. We examined the association between three distinct genotypes, wild-type (normal), heterozygous (one FVL allele), and homozygous mutated (two FVL alleles), and the activated protein C (APC) ratio. The assay was performed using ACL TOP 750 (Instrumentation Laboratory, Bedford, MA, USA), with the appropriate reagents and controls as specified in the laboratory protocols.

### 2.3. Statistical Analysis

The statistical analysis of the five polymorphic markers involved several crucial steps, including determining the frequencies of the major and minor alleles (*p* and q), performing Hardy–Weinberg Equilibrium (HWE) tests using chi-square statistics, and examining pairwise linkage disequilibrium using D′ and r^2^ metrics. Moreover, a chi-squared test of independence was performed to determine whether there was a statistically significant relationship between sex, age, and the distribution of each genetic marker.

Furthermore, a power analysis was conducted in advance to determine the sample size required to detect effect sizes (Δ) of 0.1 to 0.9 with 80% power, and a post hoc analysis confirmed the achieved statistical power verifying the robustness of the genetic study. All analyses were performed using the Python programming language (version 3.11), http://www.python.org, accessed on 1 September 2025 with libraries including Pandas (version 2.2.2), NumPy (version 1.26.0), and SciPy (version 1.12.0) along with various visualization tools.

For the FV Leiden testing and APC ratio, the statistical analysis comprised descriptive statistics (mean, standard deviation, and quartiles) for each genotype group, normality assessment using Shapiro–Wilk tests, homogeneity of variance testing using Levene’s test, and the non-parametric Kruskal–Wallis test to evaluate differences between groups, selected due to violations of normality assumptions. Additionally, threshold analysis was conducted using a clinically significant APC ratio cutoff of 2.0.

## 3. Results

Our study examined 2078 individuals aged 18–85 years (mean age: 50.5 years), with a sex distribution of 1143 males (55%) and 935 females (45%). Owing to incomplete genotyping, the sample sizes ranged from 198 for *PAI-1* to 2.078 for FV Leiden. No significant differences in age or sex were observed, which was attributable to the autosomal inheritance pattern of these variants (Figure S3). We assessed the allele frequencies and genotype distributions of five polymorphisms linked to thrombophilia: FV Leiden, *FII* (prothrombin G20210A), *MTHFR* C677T, *MTHFR* A1298C, and *PAI-1* 4G/5G. The FV Leiden (7.3%) and *FII* (3.7%) mutations showed low minor allele frequencies, typical of these variants, whereas *MTHFR* polymorphisms had higher frequencies (~33%). *PAI-1* exhibited an almost balanced allele distribution (minor allele frequency of 44%), which was notable.

Genotype distribution analysis of 1457 individuals for FV Leiden revealed that 1275 (85.7%) were wild-type (GG), 176 (13.8%) were heterozygous (GA), and six (0.4%) were homozygous mutants (AA). The global minor allele frequency (MAF) was 0.07 in the analyzed cohort.

**Figure 1:**
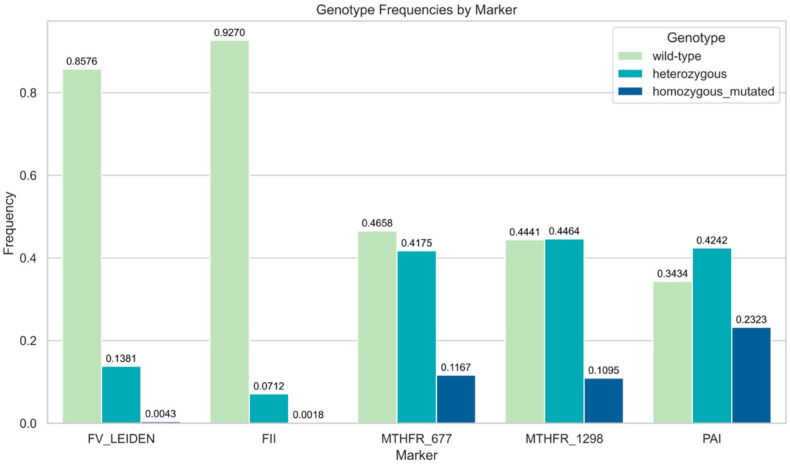
Genotype frequencies by marker.

The *FII* G20210A polymorphism was mainly wild-type, with 92.7% of the individuals studied being GG, 7.1% being heterozygous (GA), and 0.0018% being homozygous mutants, leading to a MAF of 3.7%. The *MTHFR* C677T polymorphism was more evenly distributed, with 46.5% of the samples being wild type (CC), 41.7% being heterozygous (CT), and 11.6% being homozygous mutant (TT), resulting in a MAF of approximately 33%. Similarly, the *MTHFR* A1298C polymorphism exhibited 44.4% wild-type (AA), 44.6% heterozygous (AC), and 10.9% homozygous mutant (CC) genotypes, corresponding to an MAF of 33.5%. For the *PAI-1* 4G/5G polymorphism, the genotypes were nearly evenly distributed: 34.3% wild-type (5G/5G), 42.4% heterozygous (4G/5G), and 23.2% homozygous mutant (4G/4G), yielding the highest MAF of 44% among the variants analyzed (Figure 1).

In addition, the co-inheritance of specific single-nucleotide polymorphisms (SNPs) that met the frequency thresholds and had a sufficient sample size, specifically *MTHFR* C677T and *MTHFR* A1298C, was identified in 435 individuals (24.7%), with both variants present in a heterozygous type (Figure S4). Both *MTHFR* A1298C and *PAI-1* were heterozygous in 39 patients (22.8%). Triplet co-inheritance involving FV Leiden, *MTHFR* C677T, and *MTHFR* A1298C was observed in 55 subjects (3.1%) (Table S1, Supplementary Material).

**Figure 2:**
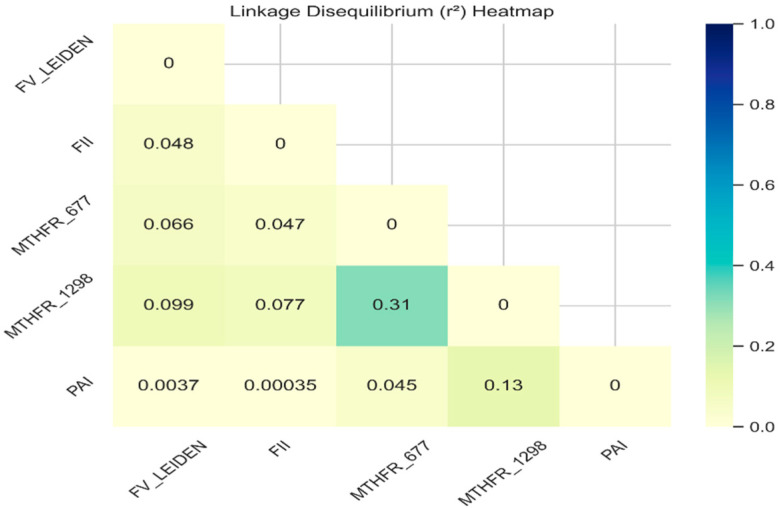
Linkage disequilibrium (r^2^ heatmap).

In addition, pairwise linkage disequilibrium (LD) analysis revealed several noteworthy co-inheritance patterns, such as *MTHFR* C677T and *MTHFR* A1298C, which showed moderate linkage (D′ = 0.5964, r^2^ = 0.3137), consistent with their physical proximity within the *MTHFR* gene (Figure 1). *FII* and *MTHFR* A1298C showed a maximum D′ (1.0000) but a low correlation (r^2^ = 0.0765), indicating limited practical co-inheritance. FV Leiden and *MTHFR* A1298C showed a notable non-random association (D′ = 0.8378, r^2^ = 0.0987). *MTHFR* A1298C and *PAI-1* exhibited the second strongest co-inheritance pattern (D′ = 0.4799, r^2^ = 0.1300). *FII* and *PAI-1* segregated almost independently (D′ = 0.0817, r^2^ = 0.0003) (Figure 2).

**Figure 3:**
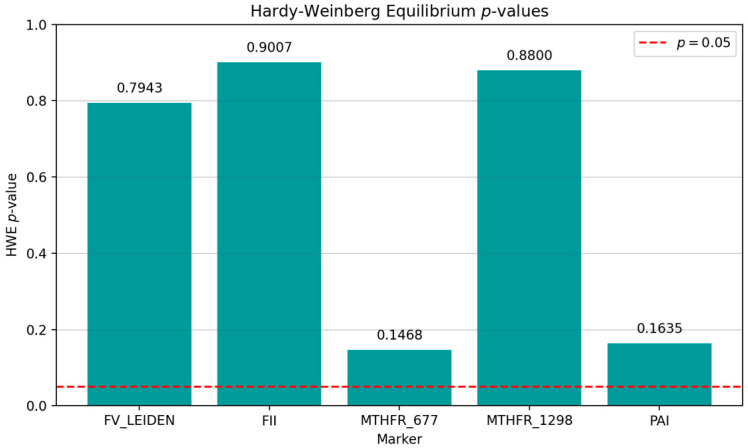
Hardy–Weinberg equilibrium *p*-values.

Concerning the Hardy–Weinberg equilibrium, FV Leiden, *FII*, *MTHFR* C677T, and *MTHFR* A1298C exhibited the following chi-square (χ^2^) and *p*-values: FV Leiden: χ^2^ = 0.07, *p* = 0.7943; *FII*: χ^2^ = 0.02, *p* = 0.9007; *MTHFR* 677: χ^2^ = 2.10, *p* = 0.1468; *MTHFR* 1298: χ^2^ = 0.02, *p* = 0.8800; and *PAI-1*: χ^2^ = 1.94, *p* = 0.1635, respectively (Figure 3) [1]. All markers maintained Hardy–Weinberg equilibrium (*p* > 0.05) (Figure 3).

FV Leiden, *FII*, *MTHFR* C677T, and *MTHFR* A1298C achieved statistical power ≥ 0.99 for effect sizes (Δ) ≥ 0.1, whereas *PAI-1* achieved 0.51 at Δ = 0.1 due to its smaller sample size (*n* = 198). The sample size for most markers was sufficiently robust to detect small effect sizes (0.1) with a high statistical power. Nonetheless, the *PAI-1* marker requires a larger sample size to improve its power to identify minor effects. Furthermore, power analysis demonstrated that FV Leiden, *FII*, *MTHFR* C677T, and *MTHFR* A1298C achieved statistical power ≥ 0.99 across all tested effect sizes (Δ = 0.1 to 0.9). *PAI-1* 4G/5G attained a power of approximately 0.51 at Δ = 0.1, with power exceeding 0.99 for Δ ≥ 0.2. This result can be attributed to the small sample size (*N* = 198). The effect sizes were classified as small (Δ = 0.1), moderate (Δ = 0.3–0.5), and strong (Δ ≥ 0.8) (Figures S2 and S3).

Among the 2078 patients evaluated for the FV Leiden mutation, 1476 also underwent concurrent testing for activated protein C resistance (APC ratio). APC ratio values differed significantly across genotypes with means of 2.71 ± 0.31 (range: 1.82–3.99) for the wild-type, 1.75 ± 0.14 (range: 1.36–2.15) for the heterozygous, and 1.25 ± 0.06 (range: 1.18–1.32) for the homozygous FVL (Kruskal–Wallis H = 475.18, *p* < 0.0001); utilizing an APC ratio threshold of <2.0, the method demonstrated an accuracy of 98.9%, along with a sensitivity of 99.2% and a specificity of 96.6%. With a minimal overlap of 1.82 to 2.15, the detection rates of both homozygous and heterozygous mutations were exceptionally high.

## 4. Discussion

Thrombophilia represents a genetic predisposition to thrombotic events shaped by population-specific allelic distributions and intricate linkage patterns. The allele frequencies were consistent with the expected distributions in population genetics studies. FV Leiden (7.3%) and *FII* (3.7%) showed low minor allele frequencies, while Methylenetetrahydrofolate Reductase (*MTHFR*) polymorphisms C677T and A1298C showed higher frequencies (32.5% and 33.2%, respectively). Plasminogen Activator Inhibitor-1 (*PAI-1*) exhibited an almost balanced allele distribution (minor allele frequency of 44%), which was notable. The *MTHFR* C677T and *MTHFR* A1298C variants showed substantial correlation, likely due to their proximity to chromosome 1. The genotype distribution did not deviate significantly from the Hardy–Weinberg equilibrium (*p* > 0.05). Furthermore, the APC ratio demonstrated high accuracy, sensitivity, and specificity in distinguishing FV Leiden genotypes.

Our results on FV Leiden align with those of Varga et al., who found that patients with a family or medical history of venous thromboembolism (VTE) had a higher prevalence of FVL. Also, Clark et al. showed heterozygosity rates up to 15–20% or higher in selected high-risk families or clinical cases. Homozygosity remains more frequent than in the general population, but is still uncommon [19]. FVL is widespread in Europe and the Eastern Mediterranean, with an allele frequency of 4.4% in healthy subjects in the Czech Republic and Turkey. At the same time, Slovenia, Croatia, and Serbia have reduced frequencies (below 2.8%, averaging 1.9%), including Spain (2.0%), Poland (2.4%), and Serbia (2.2%). The Spanoudaki et al. study in healthy subjects in Northern Greece showed an allele frequency of 3% with genotype frequencies of 6% heterozygous and 0% homozygous [20]. The relatively high MAF in Greece can be attributed to the local genetic structure, historical demographic events, and a complex mosaic shaped over multiple epochs, with further amplification in isolated groups.

The results of the prothrombin G20210A (*FII*) mutation (MAF 3.7%) align with previous studies in Northwestern Greece, in which the prevalence was 2.7% and 2.04% in the Mediterranean population of Sehilari et al., and 2% in the Greek Cypriot population 2%. In Southern Italy, the prevalence rate was reported to be 5.7% among blood donors, which is higher than the average prevalence observed in other parts of Europe [21]. Our study in patients revealed a minor allele frequency (q) of 0.07, consistent with its established prevalence in European populations. In contrast, prothrombin G20210A showed a lower frequency (q = 0.04), aligning with its secondary thrombophilic role [22]. The results of the prothrombin G20210A (*FII*) mutation (MAF 3.7%) and 7.1% being heterozygous (GA), and 0.0018% being homozygous mutant, align with the previous studies reporting 4.3–10.2% heterozygosity in (VTE) patients.

The *MTHFR* C677T and A1298C polymorphisms vary geographically across European populations, influenced by genetic architecture, lifestyle factors, and folate intake. Our findings align with Northern European distributions but show higher heterozygosity, following Southern European populations where the C677T allele reaches 35–50%, as seen in Greek and Italian cohorts. Our findings on the *MTHFR* A1298C mutation showed a higher allele frequency compared to other studies. The 1298C allele frequency ranged from 17% to 44% in Asia, 24% to 40% in Europe, 0% to 15% in South America, and 14.7% in North America. Our results align with those of Zappacosta et al. in central-southern Italy, where the C allele frequency for A1298C among newborns was 32.7%, with 12.5% homozygous (CC) and 44.6% heterozygous (AC) genotypes in the Mediterranean region [23,24,25,26,27]. Our research found differences from that of Mazokopakis et al. [28], which might be caused by regional genetic heterogeneity. Geographic barriers, such as mountains and islands, can restrict gene flow, resulting in distinct genetic profiles. This finding highlights the importance of examining genetic variations between ethnic and regional subgroups in wider geographical areas, because there are also substantial differences between apparently homogeneous groups [28].

Moreover, we found significant co-inheritance between *MTHFR* C677T and *MTHFR* A1298C (24.7%), which affects different functional regions of the same gene, leading to reduced *MTHFR* enzymatic activity. This co-inheritance leads to cumulative enzymatic dysfunction, disruption of folate metabolism, and homocysteine regulation. Genetic predisposition may be influenced by environmental, lifestyle, and dietary factors, which can affect the distribution of alleles within populations.

In addition, in our study, the *PAI-1* 4G/5G polymorphism contrasts with earlier studies that documented a lower prevalence of the 4G allele, such as the 12.4% frequency reported by Ferrara et al. in healthy Italian controls, the 49.4% frequency observed by Ringelstein et al. in the German population, and the 38% reported by Cortesao in the Portuguese population [29,30].

We analyzed linkage disequilibrium (LD), which revealed the strongest LD between the *MTHFR* C677T and A1298C variants, reflecting their proximity (621 bp apart). The second strongest co-inheritance pattern was between *MTHFR* A1298C and *PAI-1*, which was surprising given the genomic distance between markers, implying shared evolutionary pressures. The high D′ value (0.8378) between FV Leiden and *MTHFR* A1298C suggests partial co-inheritance with limited recombination, although the r^2^ value (0.0987) limits the association mapping. A similar pattern between FV Leiden and *MTHFR* C677T indicated a reduced ancestral linkage due to recombination or allele frequency disparities. *FII* and *MTHFR* A1298C exhibited complete LD, suggesting that demographic or selective factors restricted the haplotype diversity. In contrast, *PAI-1* marker pairs, (*FII–PAI-1*) and (FV Leiden–*PAI-1*), reflecting minimal co-inheritance, possibly due to chromosomal separation and recombination rates. These results emphasize the complexity and heterogeneity of LD patterns among thrombosis-related genetic markers and underscore the importance of considering population-specific haplotype structures and marker independence when conducting genetic association studies for complex traits.

All loci showed *p* > 0.05 in Hardy–Weinberg equilibrium, indicating stable populations. The differences between the observed and expected genotype frequencies were likely due to random probability. The population shows genetic stability through random mating, without selection pressure or drift. No evidence of genotyping errors, stratification, or selection bias exists. Power analyses confirmed robust detection of small effect sizes (100% power for markers). These findings underscore the need for population-tailored approaches, taking into consideration allele frequencies, genetic structure, and demographic history, to assess thrombophilia risk rather than applying a uniform model.

**Figure 4:**
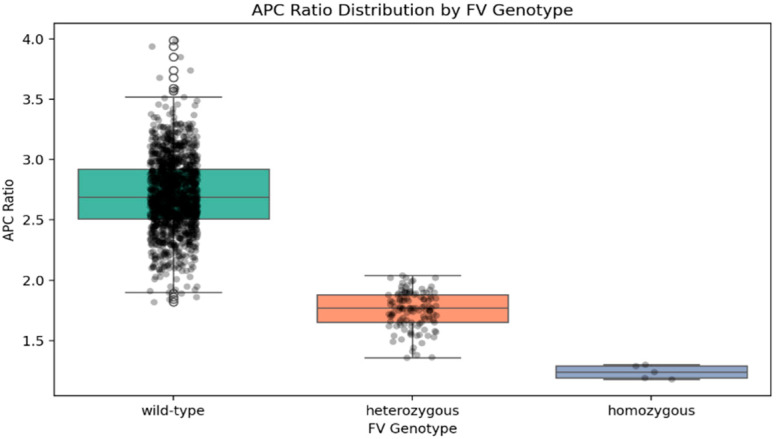
APC Ratio Distribution by FV Leiden Genotype.

The activated protein C ratio (APC ratio) showed exceptional specificity, sensitivity, and accuracy. Genetic testing is reserved for confirmation in cases in which the results impact long-term management, such as lifelong anticoagulation, family screening, or reproductive counseling. Nevertheless, genetic testing is required for definitive diagnosis in borderline cases, between 1.82 and 2.15, where wild-type and heterozygous ranges intersect, while the homozygous range remains distinct (Figure 4).

Population-based data allow clinicians to adjust thromboprophylaxis recommendations according to local genetic factors and also help clinicians identify groups with high genetic risk, especially those with venous thromboembolism in their families, enabling better risk stratification in critical scenarios such as surgery, hormone therapy, and pregnancy management. The findings can act as a benchmark for broader regional or national programs aimed at standardizing genetic screening and preventive care policies.

Our study has certain limitations. This single-center, cross-sectional study has limitations, including individuals who were able to visit the hospital in person and possibly exclude severely ill or immobile individuals. Regional focus on northern Greece and the single-focus research design may lead to population bias, which, due to relatively uniform genetic, environmental, and social and cultural traits, may limit the scope of the research’s results and therefore does not adequately represent the genetic diversity of large Greek or European populations. Additionally, technical issues like reagent quality, primer specificity, and the integrity of the DNA sample further influence the PCR results, even when strict quality controls are introduced. The cost-effectiveness of genetic screening protocols and the APC resistance test was not evaluated, as economic analysis was beyond the scope of the present study. Variable sample sizes were observed across genetic markers for the subset of *PAI-1* promoter polymorphisms. The exclusive focus on genetic data without clinical outcomes or lifestyle information constrains the findings’ interpretability and translational relevance.

A population-based study of thrombophilia polymorphisms, considering specific tested parameters, informs clinical practice, guides health policy, and supports research on screening protocols and anticoagulation strategies. These insights will improve the management and prevention of thrombotic disorders in different populations. Comparative and future multicenter research should investigate geographic and ethnic risk differences, novel mutations, and epigenetic influences, while validating genetic risk scores and incorporating pharmaceutical and lifestyle factors into risk stratification models and clinical application of the findings.

## 5. Conclusions

The estimation of allele and genotype frequencies of thrombophilia mutations (FV Leiden, *FII*, Methylenetetrahydrofolate Reductase (*MTHFR* C677T and *MTHFR* A1298C), and Plasminogen Activator Inhibitor-1 (*PAI-1*)) offers numerous advantages in different areas. These estimates inform public health screening and management strategies, particularly in populations with high carrier rates. Increased carrier prevalence contributes to a higher incidence of thrombophilia-related events, especially when additional risk factors such as immobility, surgical procedures, or estrogen use are present.

The observed linkage disequilibrium between *MTHFR* C677T and A1298C and *MTHFR* A1298C and *PAI-1* suggests coordinated genetic impacts on homocysteine metabolism and fibrinolysis. Identifying these co-inherited genetic variants may improve diagnostic accuracy and guide the customization of preventive thromboprophylaxis strategies in high-risk subgroups. The ability to combine genetic data with lifestyle habits and family history to create rule-based and algorithmic risk models is crucial for clinical practice and personalized medicine. Future large-scale comparative studies with other populations will elucidate geographic or ethnic differences in thrombophilia risk as well as the search for novel mutations and epigenetic factors, thereby deepening our understanding of the genetic architecture underlying thrombotic diseases.

## Data Availability

The data presented in this study are available in the article and Supplementary Material.

## Supplementary Materials

The following supporting information can be downloaded at: https://www.mdpi.com/article/10.3390/medicina61111935/s1, Table S1: Single Nucleotide Polymorphism (SNP) pair combination frequencies, MTHFR_677: MTHFR C677A, MTHFR_1298: MTHFR A1298C, PAI: PAI-1 4G/5G, het: heterozygous, hom: homozygous_mutated, Figure S1: Sample size needed versus effect size, Figure S2: Power achieved versus effect size, Figure S3: Sex and genetic polymorphisms, Figure S4: Genotype combination frequencies for MTHFR C677T/MTHFR A1298C.

## Abbreviations

FV LEIDEN	Factor V Leiden
FII	Factor II
MTHFR C677T	Methylenetetrahydrofolate Reductase C677T
MTHFR A1298C	Methylenetetrahydrofolate Reductase A1298C
PAI-1	Plasminogen Activator Inhibitor-1
LD	linkage disequilibrium
MAF	Minor Allele Frequency
VTE	Venous Thromboembolism
