# Supplementary material for: Population-Based Genetic Assessment of Thrombophilia Polymorphisms: Allelic Frequencies and Population Linkage Dynamics
Source: Medicina (Kaunas). 2025 Oct 29;61(11):1935. doi: 10.3390/medicina61111935 (PMC12654480; doi:10.3390/medicina61111935)
Supplement: Supplementary file 1 [file medicina-61-01935-s001.zip › medicina-3928136-supplementary.pdf]

Supplementary Material

Table S1: Single Nucleotide Polymorphism (SNP) pair combination frequencies

SNP_Pair	FV_LEIDEN	FII	MTHFR_677	MTHFR_1298	PAI	Count	Frequency
FII & MTHFR_1298		het		het		53	0,037456
FII & MTHFR_1298		het		hom		11	0,007774
FII & MTHFR_1298		hom		het		1	0,000707
FII & MTHFR_677		het	het			46	0,028117
FII & MTHFR_677		het	hom			13	0,007946
FII & PAI		het			het	4	0,020942
FII & PAI		het			hom	4	0,020942
FII & PAI		hom			het	1	0,005236
FV_LEIDEN & FII	het	het				22	0,013269
FV_LEIDEN & FII	hom	het				1	0,000603
FV_LEIDEN & MTHFR_1298	het			het		103	0,058423
FV_LEIDEN & MTHFR_1298	het			hom		29	0,016449
FV_LEIDEN & MTHFR_1298	hom			hom		3	0,001702
FV_LEIDEN & MTHFR_1298	hom			het		1	0,000567
FV_LEIDEN & MTHFR_677	het		het			104	0,052314
FV_LEIDEN & MTHFR_677	het		hom			19	0,009557
FV_LEIDEN & MTHFR_677	hom		het			2	0,001006
FV_LEIDEN & PAI	het				hom	9	0,045455
FV_LEIDEN & PAI	het				het	8	0,040404
MTHFR_1298 & PAI				het	het	39	0,22807
MTHFR_1298 & PAI				het	hom	18	0,105263
MTHFR_1298 & PAI				hom	het	7	0,040936
MTHFR_1298 & PAI				hom	hom	2	0,011696
MTHFR_677 & MTHFR_1298			het	het		435	0,2473
MTHFR_677 & MTHFR_1298			het	hom		1	0,000569
MTHFR_677 & PAI			het		het	39	0,201031
MTHFR_677 & PAI			het		hom	14	0,072165
MTHFR_677 & PAI			hom		het	9	0,046392
MTHFR_677 & PAI			hom		hom	6	0,030928

MTHFR_677: MTHFR C677A, MTHFR_1298: MTHFR A1298C , PAI: PAI-1 4G/5G, het: heterozygous, hom: homozygous_mutated

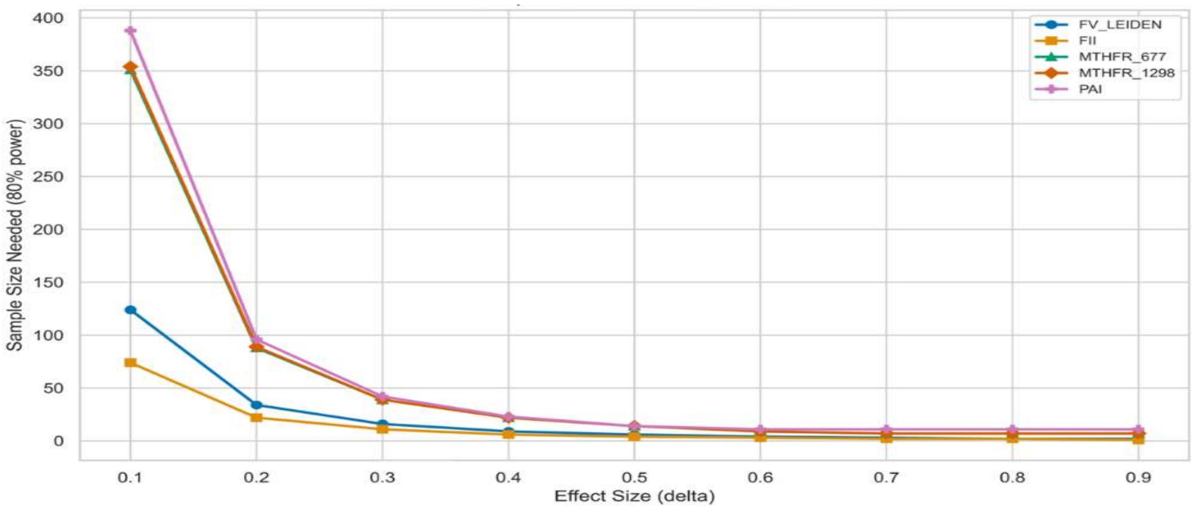

Figure S1: Sample size needed versus effect size

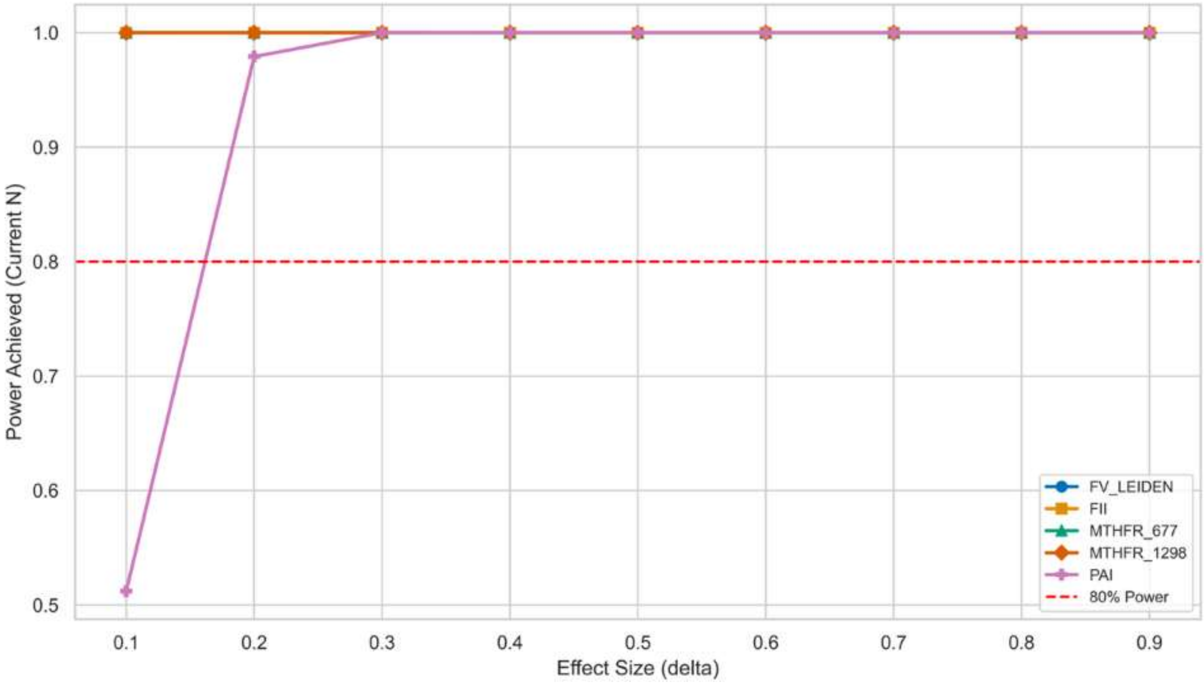

Figure S2: Power achieved versus effect size

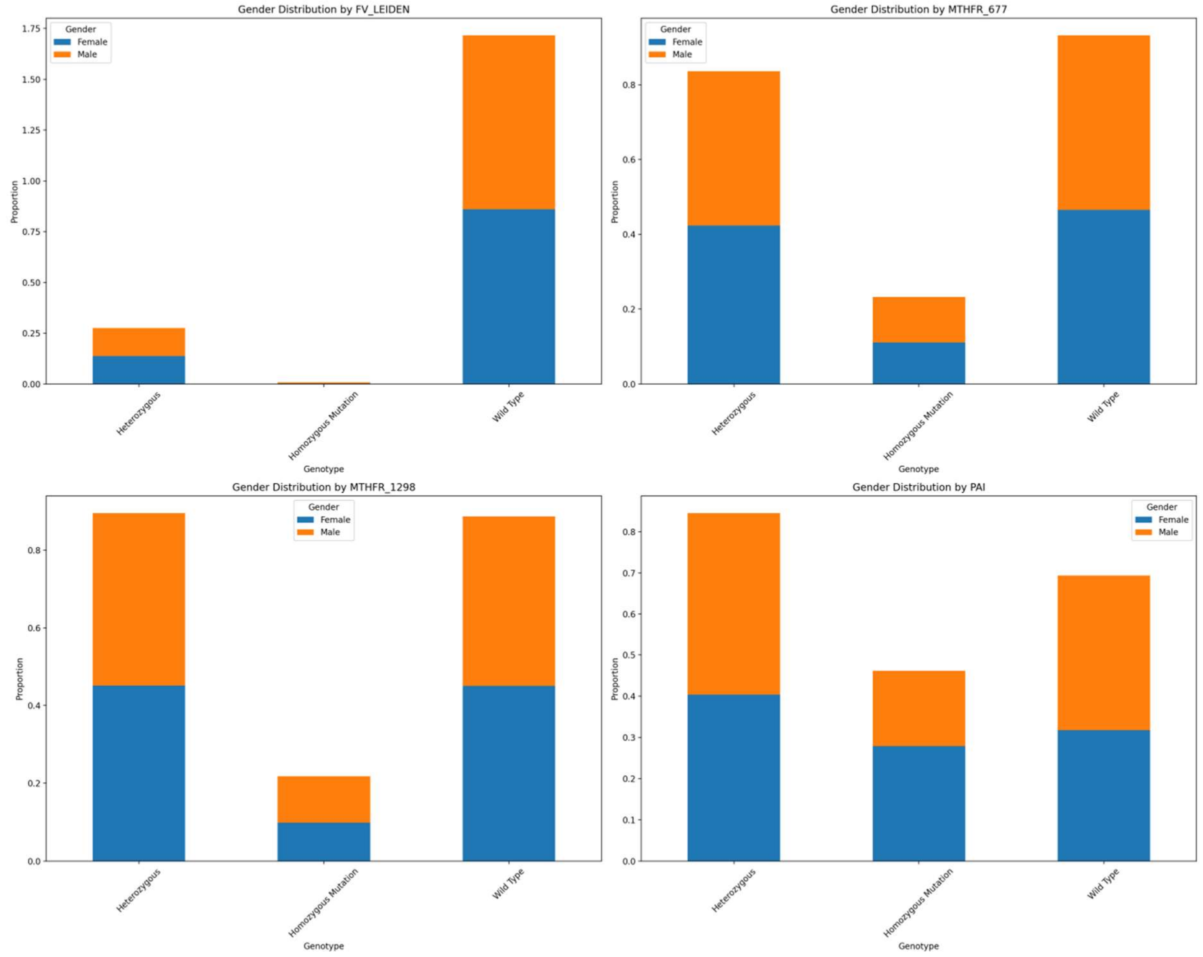

Figure S3: Sex and genetic polymorphisms

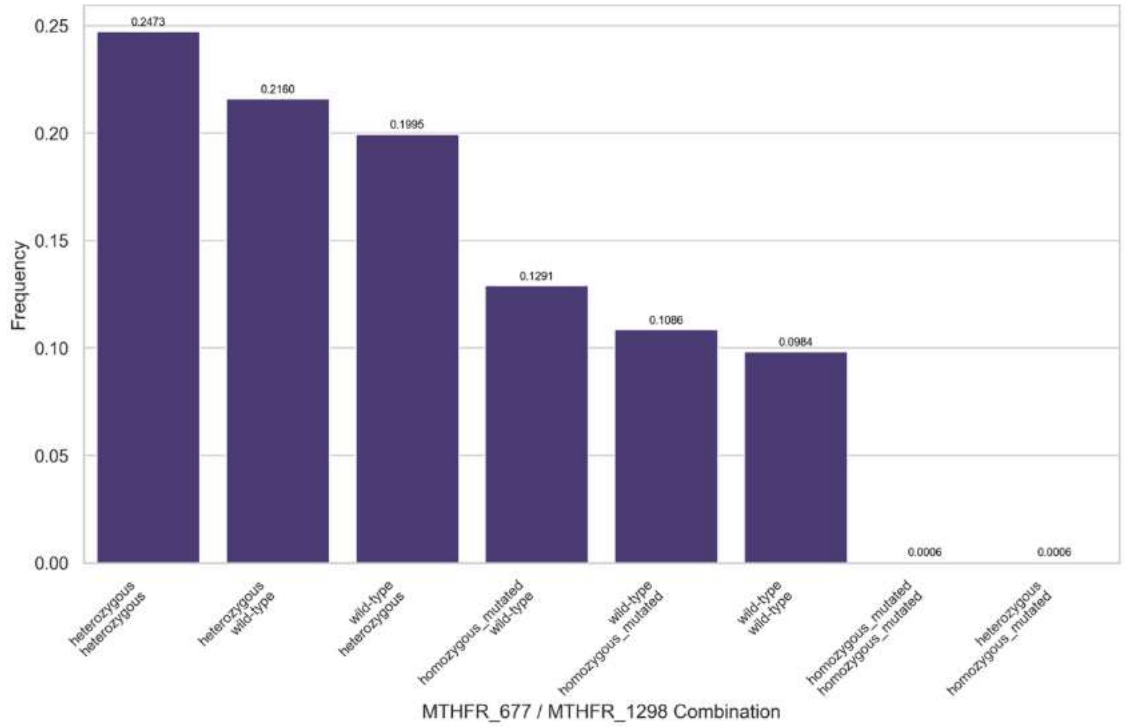

Figure S4: Genotype combination frequencies for MTHFR C677T/MTHFR A1298C
